# Supplementary material for: Could SARS-CoV-2 be transmitted via speech droplets?
Source: medRxiv. 2020 Apr 6:2020.04.02.20051177. Preprint. [Version 1] doi: 10.1101/2020.04.02.20051177 (PMC7217076; doi:10.1101/2020.04.02.20051177)
Supplement: 1 [file NIHPP2020.04.02.20051177-supplement-1.pdf]

Supplementary Information

In response to the urgency of the current pandemic, our preliminary data were recorded by a rapidly repurposed optical arrangement that employed a Coherent Verdi laser operating at 2.5 W optical power and a pair of spherical (-25-mm f.l.) and cylindrical (40.6-mm f.l.) lenses to generate a light 'sheet' approximately 1-mm thick and 150-mm tall. This light sheet passed through slits on the sides of a cardboard box (53 cm width; 46 cm height; 62-cm depth) whose interior was painted black. The box was positioned under a HEPA filter to eliminate scattering from dust particles. When speaking through the open end of the box, speech droplets traversed approximately 50-75 mm before encountering the light sheet. An iPhone 11 Pro video camera viewed the light sheet through a 7-cm diameter hole on the opposite side of the box and recorded sound and video of light scattering events as droplets passed through this sheet. Python software was developed to analyze frame-by-frame the movie clips recorded. Video clips acquired while speaking with and without a face mask are available at https://doi.org/10.5281/zenodo.3732625.
